# Determinants of Catalan Public Primary Care Professionals’ Intention to Use Digital Clinical Consultations (eConsulta) in the Post–COVID-19 Context: Mixed Methods Study

**DOI:** 10.2196/28944

**Published:** 2021-06-24

**Authors:** Francesc Saigí-Rubió, Josep Vidal-Alaball, Joan Torrent-Sellens, Ana Jiménez-Zarco, Francesc López Segui, Marta Carrasco Hernandez, Xavier Alzaga Reig, Josep Maria Bonet Simó, Mercedes Abizanda González, Jordi Piera-Jimenez, Oscar Solans

**Affiliations:** 1 Faculty of Health Sciences Universitat Oberta de Catalunya Barcelona Spain; 2 Interdisciplinary Research Group on ICTs Universitat Oberta de Catalunya Barcelona Spain; 3 Health Promotion in Rural Areas Research Group Gerencia Territorial de la Catalunya Central Institut Catala de la Salut Sant Fruitos de Bages Spain; 4 Unitat de Suport a la Recerca de la Catalunya Central Fundacio Institut Universitari per a la Recerca a l'Atencio Primaria de Salut Jordi Gol i Gurina Sant Fruitos de Bages Spain; 5 Faculty of Medicine University of Vic - Central University of Catalonia Vic Spain; 6 Faculty of Economics and Business Universitat Oberta de Catalunya Barcelona Spain; 7 Centre de Recerca en Economia i Salut Pompeu Fabra University Barcelona Spain; 8 Northern Metropolitan Primary Care Directorate Institut Català de la Salut Badalona Spain; 9 Barcelona Primary Care Directorate Institut Català de la Salut Barcelona Spain; 10 Health Department Catalan Ministry of Health Barcelona Spain; 11 Pere Virgili Health Park Primary Care Management Control Barcelona Spain; 12 Digitalization for the Sustainability of the Healthcare System Sistema de Salut de Catalunya Barcelona Spain; 13 Servei Catala de la Salut Barcelona Spain; 14 Open Evidence Research Group Universitat Oberta de Catalunya Barcelona Spain

**Keywords:** COVID-19, teleconsultation, eConsultation, eHealth, intention to use, digital health, Technology Acceptance Model, TAM, remote consultation, telemedicine, digital technology, intention, technology assessment, telehealth, pandemic, digital tool

## Abstract

**Background:**

Telemedicine has become a necessary component of clinical practice for the purpose of providing safer patient care during lockdowns due to the COVID-19 pandemic. It has been used to support the health care needs of patients with COVID-19 and routine primary care patients alike. However, this change has not been fully consolidated.

**Objective:**

The objective of this study was to analyze the determinants of health care professionals’ intention to use the eConsulta digital clinical consultation tool in the post–COVID-19 context.

**Methods:**

A literature review of the Technology Acceptance Model allowed us to construct a theoretical model and establish a set of hypotheses on the influence of a variety of different factors relating to health care professionals, as well as the institutions where they work, on their intention to use eConsulta. In order to confirm the proposed model, a mixed qualitative and quantitative methodology was used, and a questionnaire was designed to serve as the data collection instrument. The data were analyzed using univariate and bivariate analysis techniques. To confirm the theoretical model, exploratory factor analysis and binary logistic regression were applied.

**Results:**

The most important variables were related to perceived benefits (*B*=2.408) and the type of use that individuals habitually made of eConsulta (*B*=0.715). Environmental pressure (*B*=0.678), experience with technology (*B*=0.542), gender (*B*=0.639), and the degree to which eConsulta had been implemented (*B*=0.266) were other variables influencing the intention to use the tool in the post–COVID-19 context. When replicating the previous analysis according to professional group, experience with technology and gender in the physician group, and experience with tool use and the center where a professional worked in the nurse group, were found to be of considerable importance.

**Conclusions:**

The implementation and use of eConsulta had increased significantly as a consequence of the COVID-19 pandemic, and the majority of health care professionals were satisfied with its use in practice and planned to incorporate it into their practices in the post–COVID-19 context. Perceived benefits and environmental pressure were determining factors in their attitude toward and intention to use eConsulta.

## Introduction

### Background

Lockdowns and social distancing in response to the high rate of COVID-19 transmission have become the main triggers of a challenging digital transformation in many sectors, especially in health care. In this scenario of extreme crisis, the rapid adoption of digital solutions and technological tools has played an important role in the response to the huge pressure on health care systems [1-3]. Telemedicine has become a necessary component of clinical practice for the purpose of providing safer patient care [4,5], and it has been used to support the health care needs of patients with COVID-19 and routine primary care patients alike [6-10].

While the digital transformation in health care has not been as disruptive as the transformations observed in other industries, the spread of COVID-19 seems to have provided a solid and inevitable reason to fully adopt the digital transformation [11]. However, despite the fact that health care services can largely be provided remotely via digital technologies [12], this change has not become fully consolidated [13,14]. This means that further research contributions are still required in relation to the definition and adoption of new digital care models.

To establish telemedicine in routine health care, acceptance by users—health care professionals—is of vital importance to the effective use of technological resources. A variety of factors may explain why a group adopts a specific technological tool to a lesser or greater extent [15]. Of these, social factors are perceived to be the most complex. Legal limitations, patient indifference, lack of remuneration, and uncoordinated implementation by those responsible for formulating policies are weighty arguments explaining stakeholders’ refusal to engage [15,16]. Some studies have noted that these difficulties may be due to a lack of focus in the implementation of such interventions (ie, health care professionals do not see them as either necessary or effective [17]), or the paucity or inconclusive nature of the studies published thus far [18-20].

To understand whether and how the digital technologies adopted to cope with the COVID-19 crisis will continue to be useful in the postemergency phase—beyond research into outcomes thereof (efficiency, care service quality, etc)—it is necessary to understand the determinants of their use. This paper presents an ex-ante analysis and aims to provide evidence on the determinants of use of the Catalan public health care system’s eConsulta tool.

eConsulta, which forms part of a personal health folder [21], is an asynchronous teleconsultation tool available to the 7.5 million inhabitants of Catalonia (located in northeastern Spain) and to its primary care professionals. It was launched in 2015 to complement face-to-face care. The tool’s implementation has gradually been extended to the entire primary care network (more than 92% of primary care teams [PCTs] have used the tool at some point), and it has recently begun to be introduced into the public hospital setting. However, its rate of use up to March 2020 was low compared to face-to-face consultations (just 0.9% of the total) [22]. Previous studies of telemedicine acceptance by the Catalan public system’s health care professionals have suggested that despite being rated positively, especially by nursing staff, the potential technical or organizational disadvantages of the tools were negative predictors of their use [23].

The objective of this study is, therefore, to analyze the determinants of Catalan public primary care professionals’ intention to use the eConsulta digital clinical consultation tool in the post–COVID-19 context. These health care professionals (physicians and nurses) work for the Catalan Health Institute (Institut Català de la Salut [ICS]), the main provider of primary care services in Catalonia (providing a 74% coverage of the Catalan population).

### Hypotheses and Model

Regarding the theoretical approach, the Technology Acceptance Model (TAM) was used [24,25]. Uptake and use of technological applications in the health care area can be formulated as an acceptance intention, and it is therefore possible to take the TAM approach. In TAMs, technology acceptance is considered a determinant of technology use. Hence, health care professionals’ acceptance of a digital technology can be considered a determinant of its use [26-30]. Although TAMs have been widely used to explain the use of many different technologies, health care research has shown them to be highly suited to analyzing eHealth use. Following the TAM methodology, the proposed model contrasts health care professionals’ intention to use a digital tool according to two main dimensions: (1) the perceived benefits of the tool’s use and (2) its ease of use. Regarding health care professionals’ perceived benefits of the tool, the study distinguishes between those connected with improved efficiency in their care activities (better care management or provision, and time savings or reductions [31,32]), and those connected with improved quality in relation to patients [33]. Regarding ease of use, it has been shown that when a health care professional feels that a technological tool is easy to use and does not require any additional training or specific competencies, the intention to use it clearly increases [34]. Within the specific context of the eConsulta digital tool, the first two research hypotheses are:

H1. The perceived benefits of eConsulta use have an influence on the intention to use it in the post–COVID-19 context.

H2. eConsulta’s ease of use has an influence on the intention to use it in the post–COVID-19 context.

However, despite its generalized use for the purpose of corroborating health care professionals’ use of digital tools, the TAM methodology has attracted some criticism, mainly stemming from the fact that it does not take into account the influence of other types of external variable that could increase its explanatory power [35,36]. In this respect, various methodological proposals such as the theory of reasoned action and the theory of planned behavior have noted the appropriateness of considering the external influence exerted by health care professionals’ close contacts or environment [37,38]. In particular, it has been found that patients can exert pressure on a professional by asking him or her to use (or not to use) a tool [39]. Likewise, colleagues and other professional groups working in close collaboration may exert a social influence in relation to the tool’s use, either because they are users within the same organizational area or because its use has been directly recommended by them [40,41]. Lastly, the organization in which a professional works can have a direct or indirect influence on the tool’s use [29]. Thus, the organization itself can encourage its use by establishing policies and offering training, and by increasing the recognition of, or compensating, those professionals who decide to use it:

H3. Pressure from other groups (patients, health care professionals, the institution’s management team) has an influence on the intention to use eConsulta in the post–COVID-19 context.

A professional’s intention to use the tool will also be affected by professional and demographic variables [42,43]. Thus, an individual’s professional profile (physician or nurse) will determine his or her interest in, and use of, the tool. Specifically, an individual’s professional profile determines whether the tool is used to care for patients or to carry out management activities. This indirectly means that the type of pressure exerted by the environment to ensure a tool is used, and even the perceived benefits and ease of use of the tool, may be different:

H4. The professional profile has an influence on the intention to use eConsulta in the post–COVID-19 context.

Moreover, the amount of time a professional spends on doing his or her job has an influence on the intention to use the tool. Being older and more professionally experienced may have an influence on the ability or desire to learn about and use technology. Being in a certain occupational category with responsibilities may also have an influence:

H5. An individual’s experience as a health care professional has an influence on the intention to use eConsulta in the post–COVID-19 context.

H6. A health care professional’s age has an influence on the intention to use eConsulta in the post–COVID-19 context.

Similarly, gender has an influence on the intention to use the tool, mainly because there is a gender bias among professionals. In particular, there is a high percentage of women in the nurse group and, among general practitioners, the distribution of men and women varies as the population gets younger, due to the gradual feminization of the medical profession since the end of the 20th century. By the beginning of the 21st century, 70% of all new medical students were women. Since then the figure has risen to 85% [44,45]:

H7. A health care professional’s gender has an influence on the intention to use eConsulta in the post–COVID-19 context.

Lastly, the health care center where a professional works also determines the intention to use a tool, mainly for reasons of resource availability and management policies at the center:

H8. The health care region or zone in which the health care center where a professional works is located has an influence on the intention to use eConsulta in the post–COVID-19 context.

H9. The degree of eConsulta implementation at the health care center where a professional works has an influence on the intention to use it in the post–COVID-19 context.

H10. The ease of use of eConsulta (current version) has an influence on the intention to use it in the post–COVID-19 context.

**Figure 1:**
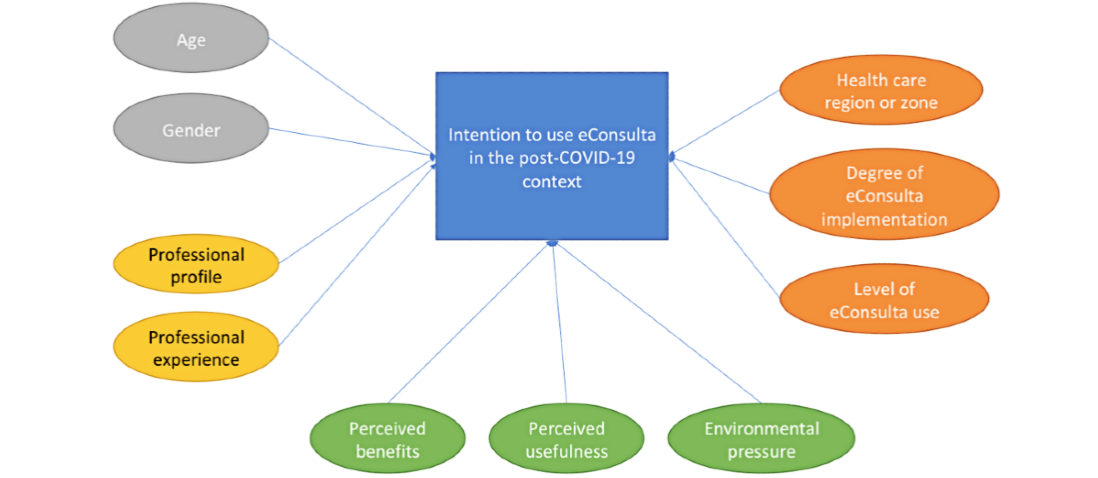
Proposed model of health care professionals’ intention to use the eConsulta tool in the post–COVID-19 context.

In summary, we consider that the health care professionals’ intention to use the tool in the post–COVID-19 context depends on four main groups of variables: (1) their perception of the tool, (2) external pressure, (3) their profiles, and (4) an additional set of factors linked to the health care center where they work. The proposed model in this regard is shown in Figure 1.

## Methods

### Study Design and Sample Selection

As an exploratory study focusing on the analysis of a single health care institution, a mixed qualitative and quantitative methodology was used, and a questionnaire was designed to serve as the data collection instrument (Multimedia Appendix 1). A review of the literature, together with the health care professionals’ experience, served as the basis to create the study variables and the metrics used in the first version of the instrument. The measurement instrument was validated following a pretest.

The final questionnaire was organized into four blocks of questions: (1) sociodemographic and professional background, (2) tool use, (3) tool use motivations, and (4) perceived benefits of tool use.

A health care provider distributed the online questionnaire to physicians, nurses, reproductive and sexual health service staff, social workers, and client care staff working in the ICS’s various PCTs across Catalonia. Sample selection was random. The questionnaire was sent to all professionals forming the study universe. Participation was voluntary, and no incentives were offered to fill in and return the questionnaire.

A literature review of TAMs allowed us to establish the theoretical model shown in Figure 1. From this, we derived a set of hypotheses about the influence that a variety of different factors relating to both health care professionals and the institutions where they work has on those professionals’ intention to use eConsulta. All the variables shown in the hypotheses, which have an influence on the intention to use eConsulta, are described in Table 1.

The data obtained from the sample of health care professionals were analyzed using SPSS Statistics 20 software (IBM Corp). Univariate and bivariate analysis techniques were used. In order to validate the proposed hypotheses and, therefore, to confirm the theoretical model, several multivariate techniques were applied, such as explanatory factor analysis and binary logistic regression.

The study protocol was approved by the University Institute for Primary Care Research Jordi Gol Healthcare Ethics Committee (code 20/026-P).

**Table 1:** Model variables

Variable	Description
Intention to use eConsulta in the post–COVID-19 context	Dichotomous variable: 0=no, 1=yes
Perceived benefits	Metric variable indicating a professional’s degree of perceived benefit from eConsulta use. This variable was created using exploratory factor analysis
Ease of use	Variable metric indicating the degree of eConsulta’s ease of use. This variable was created using exploratory factor analysis
Environmental pressure	Variable metric indicating the degree to which a professional perceives that other agents within his or her environment (organization, patients, or colleagues) have an influence on his or her use of eConsulta. This variable was created using exploratory factor analysis
Professional profile	Categorical variable indicating an individual’s professional profile, where 1=general practitioner, 2=pediatrician, 3=family nurse, and 4=reproductive and sexual health service nurse
Professional experience	Categorical variable indicating the amount of time a professional has worked in the health care sector, where 1=less than 1 year, 2=2-5 years, 3=6-10 years, 4=11-20 years, and 5=more than 20 years
Gender	Dichotomous variable indicating an individual’s gender: 1=man, 2=woman
Age	Categorical variable indicating an individual’s age range (in years), where 1=19-29, 2=30-39, 3=40-49, 4=50-59, and 5=60 or above
Health care region or zone in which a professional works	Categorical variable indicating the health care region or zone in which a professional works
Degree of eConsulta implementation	Categorical variable indicating the degree of eConsulta implementation, where 1=not yet fully implemented, 2=fully implemented, and 3=implemented due to COVID-19
Level of eConsulta use	Categorical variable indicating how eConsulta was used, where 1=no use, 2=management use, and 3=management and consultation use

## Results

The target sample comprised 18,804 health care professionals working in 285 PCTs. The study included responses from 1189 professionals who had agreed to participate between July 6 and July 31, 2020, the period during which the questionnaire was distributed (margin of error=2.8%; 95%, p=q=0.05).

### Sample Profile

Regarding age, we found that the majority of the respondents were over 40 years old. The mean age was 48 years. We found that 33.4% (n=397) were 40-49 years, and 29.2% (n=348) were 50-59 years. It is worth noting that 15.8% (n=188) were over 60 years old, and 16.8% (n=200) were under 30 years old.

The vast majority of the respondents (n=944, 79.2%) were women, meaning that just 20.8% (n=244) were men. In terms of professional profile, 62.2% (n=739) were physicians, with various profiles, and 35.2% (n=418) were other health care professionals—nurses, matrons, or social care workers. Lastly, just 2.2% (n=23) fell into the client care staff category.

In relation to the total, and focusing on the physician group, 43.4% (n=515) were primary care physicians, and 14.8% (n=175) were pediatricians. Regarding the nonphysician health care staff, 26.8% (n=318) of the total were nurses and 7.6% (n=90) were matrons. The majority of these professionals worked in one of three zones of the Barcelona health care region: Metropolitan North (n=250, 21%), Barcelona City (n=218, 18.3%), and Metropolitan South (n=133, 11.2%). Respondents also worked in the health care regions Girona (n=213, 17.9%) and Catalonia Central (n=174, 14.6%).

The majority of the respondents had considerable experience as health care professionals. Indeed, 47.4% (n=563) had over 20 years’ experience, and 32.7% (n=390) had 11-20 years’ experience. Having gained their experience at several centers, just 48% (n=563) stated that they had spent more than 11 years in the same post (more than 20 years: n=226, 19%; 11-20 years: n=354, 29.7%). Of the remaining respondents, 28% (n=333) had spent 2-5 years in the same post.

Focusing on the workplace, the degree of eConsulta implementation was quite high. Of the total respondents, 44.9% (n=531) indicated that the tool had been fully implemented, and 13.9% (n=164) reported that it had been largely implemented. It is worth noting that 38.6% acknowledged that the pace of the tool’s implementation had quickened because of COVID-19.

Regarding the respondents’ use of eConsulta, it was found that 60.3% (n=717) had just started using it, and that 26.4% (n=314) used it regularly and intensively. In fact, 40.5% (n=418) acknowledged that they used it at least once a week, and 45.8% (n=473) used it daily. Moreover, the majority of the respondents (n=828, 69.6%) used it to carry out consultations or to appraise test results and make diagnoses, and 16.9% (n=201) acknowledged that they used it for management processes only.

Lastly, it should be noted that the impact of COVID-19 on the tool’s use was high. Of the total respondents, 38.6% (n=458) stated that the tool had been implemented in their workplace. On the other hand, 85.7% (n=1018) of respondents agreed or strongly agreed with continuing to use it in the post–COVID-19 context.

### Intention to Use eConsulta

In order to identify the factors that have an influence on eConsulta adoption, a principal component exploratory factor analysis was performed. This statistical technique allows the underlying dimensions, constructs, or latent variables of the variables observed in the study to be explored with greater precision. It was on that basis that we endeavored to explain the process of eConsulta adoption.

The values obtained from the statistical tests carried out showed the suitability of the technique employed (Kaiser-Meyer-Olkin index of sampling adequacy=0.928; approximate *χ^2^*=4122.650; Bartlett test of sphericity=171, *P*<.001) (Table 2). Three factors with an eigenvalue higher than 1 were obtained from the analysis, which in total explained 53% of the variance. Likewise, it should be noted that the rotation employed in this case was orthogonal (Varimax) because it was deemed that the different factors might not display any correlation.

**Table 2:** Rotated component matrix.^a^

Original variables	Perceived usefulness	Environmental pressure	Ease of use (experience with technology)
I feel that eConsulta is useful for managing the calendar with patients, making it easier to schedule visits at times that suit them	0.687	—^b^	—
I feel that it improves the outcomes of my care activities	0.791	—	—
It allows me to offer patients better treatment	0.752	—	—
I talk to other professional colleagues about the benefits of using eConsulta	0.718	—	—
eConsulta has been very useful to me during the COVID-19 pandemic because it has allowed me to care for patients remotely, thus reducing the risk of infection	0.586	—	—
I positively rate the potential benefits that eConsulta use can offer, both for the patients and the PCT^c^/service	0.815	—	—
I promote eConsulta use among my patients	0.744	—	—
As a result of eConsulta implementation in the PCT/service, ways of working have changed, or new ones have been introduced, at individual and group levels	0.632	—	—
The changes made to eConsulta in the COVID-19 pandemic context have made it easier to use	0.536	—	—
I feel that eConsulta is very useful for carrying out my professional activities	—	0.614	—
My colleagues use it often	—	0.662	—
The PCT/service that I work in encourages and facilitates eConsulta use	—	0.514	—
Some of my patients ask me to use it	—	0.541	—
Care professionals can access eConsulta very easily	—	0.629	—
Citizens can access eConsulta very easily	—	0.730	—
I am a habitual user of technology (both professionally and personally)	—	—	0.726
I am a habitual user of social media (both professionally and personally)	—	—	0.798
I have previous experience using telemedicine systems	—	—	0.660
Cronbach alpha	0.916	0.819	0.747
Eigenvalue	7.249	1.534	1.363
Variance explained (%)	28.4	15.3	9.6

^a^Rotation method: Varimax with Kaiser normalization.

^b^Not applicable.

^c^PCT: primary care team.

Three factors emerged from the analysis. The first explained 28.4% of the variance and included the various benefits of eConsulta use, as observed by the professionals. These benefits referred to improvements in the professionals’ relationships with patients and in the efficiency of their work. The second factor explained 15.3% of the variance and showed the pressure that third parties—work colleagues, patients, or the institution itself—directly or indirectly exerted on the professionals. Lastly, the third factor was perceived ease of use. This factor explained 9.6% of the variance and showed that some of the professionals who decided to use eConsulta were those who had previous experience of using ICTs (information and communications technologies) (personally) and even telemedicine. A confirmatory factor analysis confirmed the results obtained from the exploratory factor analysis. The results show a goodness of fit (NFI [normed fit index]= 0.897, CFI [comparative fit index]=0.906, TLI [Tucker Lewis index]=0.870, RMSEA [root mean square error of approximation]=0.085).

In order to analyze the influence that these and other factors had on the health care professionals’ intention to use eConsulta in the post–COVID-19 context, several logit analyses were performed.

### Intention to Use eConsulta in the Post–COVID-19 Context

The various analyses performed showed the model’s goodness of fit (Wald=467.731, *P*<.001; Hosmer-Lemeshow=8.525, *P*=.38). Likewise, the model displayed high explanatory power, with a Nagelkerke R^2^ value of 0.615.

Regarding the variables in the model, it was found that most of them displayed a direct and significant relationship with the intention to use eConsulta in the post–COVID-19 context (Table 3). The most important variables were those referring to perceived benefits (*B*=2.408) and the type of use that individuals *B*=0.715). Environmental pressure also contributed to the intention to continue using the tool (*B*=0.678), as did experience of using technological tools, some of which were specific to the health care area (*B*=0.542). Lastly, gender had an influence on the intention to use eConsulta (*B*=0.639), as did the degree of its implementation at the health care center where a professional works (*B*=0.266).

**Table 3:** Logistic regression on eConsulta predictors.

Variable	*B*	SE	Wald	*df*	*P* value	Exp(B)
Age	0.076	0.159	0.227	1	.63	1.079
Gender	0.639	0.308	4.303	1	.04	1.895
Professional profile	–0.280	0.103	7.438	1	.006	0.755
Health care region or zone in which a professional works	0.062	0.048	1.683	1	.20	0.940
Professional experience in the health care area	–0.022	0.164	0.018	1	.89	0.978
Benefits	2.408	­–0.199	146.029	1	<.001	11.113
Environmental pressure	0.678	0.129	27.649	1	<.001	1.971
Ease of use	0.542	0.129	17.559	1	<.001	1.720
Degree of eConsulta implementation	0.266	0.149	3.168	1	.08	1.304
Level of eConsulta use	0.715	0.176	16.469	1	<.001	2.045
Constant	0.989	0.894	1.223	1	.27	2.689

The negative value displayed by the profile of the health care professional stands out (*B*=–0.280). This would suggest that the types of activity that different groups carry out within an organizational area had an influence on the rating of, and the intention to use, eConsulta.

With that in mind, and with the aim of confirming or rejecting all the previously proposed hypotheses contained in the model, we replicated the previous analysis for two groups working in the family medicine area: general practitioners and family nurses (there was a total of 507 general practitioners, of whom 89.7% [n=455] used eConsulta and 10.3% [n=52] did not. There were 296 nurses, of whom 87.2% [n=258] used the tool and 12.8% [n=36] did not). Both groups care for adult patients, the majority of whom are advanced in years and are diagnosed with a chronic illness. They are patients whose knowledge and use of ICTs is low.

Considering the different models by group, we found that, among the general practitioner group, all the variables had a direct and positive effect on the intention to use eConsulta. This group was formed by 30.8% (n=156) men and 69.2% (n=351) women, with a mean age (in years) between 40 and 59 years (40-49 years: n=191, 37.7%; 50-59 years: n=151, 29.8%).

Analyzing Table 4, the variable with the greatest weight in the model was perceived benefits (*B*=2.472), followed by gender (*B*=1.011) and the type of use that individuals made of eConsulta (*B*=0.809). Regarding type of use, we found that 92.1% of the physicians made wide use of the tool. Environmental pressure was an important factor in the decision to use it (*B*=0.773). Lastly, an individual’s experience of technology (*B*=0.724), together with the degree of eConsulta implementation in his or her workplace (*B*=0.671), also had an influence on the intention to use the tool.

**Table 4:** Logistic regression on eConsulta predictors (general practitioners).

Variable	*B*	SE	Wald	*df*	*P* value	Exp(B)
Age	0.257	0.310	0.690	1	.41	1.293
Gender	1.011	0.485	4.353	1	.04	2.749
Health care region or zone in which a professional works	0.066	0.096	0.482	1	.49	1.069
Professional experience in the health care area	0.118	0.290	0.166	1	.68	1.126
Benefits	2.472	0.330	56.054	1	<.001	11.850
Environmental pressure	0.773	0.221	12.190	1	<.001	2.166
Ease of use	0.724	0.220	10.864	1	.001	2.062
Degree of eConsulta implementation	0.671	0.332	4.085	1	.04	1.956
Level of eConsulta use	0.809	0.451	3.220	1	.07	2.246
Constant	–2.743	1.958	1.962	1	.16	0.064

The family nurse group (Table 5) was formed by 88.6% (n=285) women, with a mean age between 40 and 59 years (40-49 years: n=104, 33%; 50-59 years: n=95, 30%). Among the variables determining their intention to use eConsulta were, in first place, perceived benefits (*B*=2.100), followed by the use they make of it (*B*=1.362). Here, we found that 50.5% (n=160) stated that they made wide use of the tool, whereas 30% (n=96) only did so for management purposes (Multimedia Appendix 2). Lastly, environmental pressure was a fundamental factor (*B*=0.479). It is worth noting that the health care region or zone in which a health care professional works displayed a value of little importance, which was negative in the model (*B*=–1.91). Of the total cases, 32% (n=101) and 11.8% (n=37) of these professionals worked in the health care zones Metropolitan North and Barcelona City, respectively (Multimedia Appendix 3).

Finally, Table 6 shows the significant variables for the three calculated models.

A comparison of the different models shows that, in all of them, perceived benefits was the variable that had the highest explanatory power. After these, in descending order of importance, were experience of eConsulta use and, lastly, the influence that patients, colleagues, and the institution itself had on the professionals. Regarding these variables, it is worth noting the considerable importance that experience with eConsulta use had for the nurse group (*B*=1.362), compared to the values displayed by this variable in the physician group (*B*=0.809) or, indeed, in the overall model (*B*=0.715).

Meanwhile, some variables were significant for the overall model but were not for the partial models. This was the case for experience with technology, which had a value of *B*=0.724 in the physician group but was not significant in the nurse group. The situation regarding the gender variable was similar; in the physician group, the variable was found to be significant and had a high B value (*B*=1.011), whereas it was not significant in the nurse group.

**Table 5:** Logistic regression on eConsulta predictors (nurses).

Variable	*B*	SE	Wald	*df*	*P* value	Exp(B)
Age	–0.293	0.316	0.860	1	.35	0.746
Gender	–0.813	0.887	0.840	1	.36	0.444
Health care region or zone in which a professional works	–0.191	0.096	4.001	1	.045	0.826
Professional experience in the health care area	–0.113	0.350	0.105	1	.75	0.893
Benefits	2.100	0.390	29.048	1	<.001	8.169
Environmental pressure	0.479	0.287	2.783	1	.10	1.615
Ease of use	0.161	0.255	0.397	1	.53	1.174
Degree of eConsulta implementation	–0.053	0.309	0.029	1	.87	0.949
Level of eConsulta use	1.362	0.367	13.791	1	<.001	3.906
Constant	3.747	1.909	3.851	1	.05	42.380

**Table 6:** Significant variables for the three calculated models.

Variable	Overall model		General practitioners		Family nurses	
	*B*	*P* value	*B*	*P* value	*B*	*P* value
Age	0.076	.63	0.257	.41	–0.293	.35
Gender	0.639	.04	1.011	.04	–0.813	.36
Professional profile	–0.280	.006				
Health care region or zone in which a professional works	0.062	.20	0.066	.49	–0.191	.045
Professional experience in the health care area	–0.022	.89	0.118	.68	–0.113	.75
Benefits	2.408	<.001	2.472	<.001	2.100	<.001
Environmental pressure	0.678	<.001	0.773	<.001	0.479	.10
Ease of use	0.542	<.001	0.724	.001	0.161	.53
Degree of eConsulta implementation	0.266	.08	0.671	.04	–0.053	.87
Level of eConsulta use	0.715	<.001	0.809	.07	1.362	<.001
Constant	0.989	.27	–2.743	.16	3.747	.05

Finally, it should be mentioned that the health care region or zone in which a professional works was a variable that displayed a negative effect on the nurse group’s intention to use eConsulta (Table 7).

Table 8 shows the hypotheses that were confirmed or rejected for each of the three models.

**Table 7:** Distribution of the family nurse group by health care region or zone.

Health care region or zone	Nurses, n (%)	Cumulative %
Metropolitan North	45 (15.2)	15.2
Central Catalonia	39 (13.1)	28.3
Barcelona City	56 (18.9)	47.1
Lleida	12 (4.0)	51.2
Metropolitan South	38 (12.8)	64.0
Girona	58 (19.5)	83.5
Camp de Tarragona	24 (8.1)	91.6
Terres de l’Ebre	25 (8.4)	100.0
Total	297 (100.0)	—^a^

^a^Not applicable.

**Table 8:** Hypotheses and results.

Hypotheses	Overall model	Physicians	Nurses
H1. The perceived benefits of eConsulta use have an influence on the intention to use it in the post–COVID-19 context.	Yes	Yes	Yes
H2. eConsulta’s ease of use has an influence on the intention to use it in the post–COVID-19 context.	Yes	Yes	No
H3. Pressure from other groups (patients, health care professionals, the institution’s management team) has an influence on the intention to use eConsulta in the post–COVID-19 context.	Yes	Yes	Yes
H4. The professional profile has an influence on the intention to use eConsulta in the post–COVID-19 context.	Yes	—^a^	—
H5. An individual’s experience as a health care professional has an influence on the intention to use eConsulta in the post–COVID-19 context.	No	No	No
H6. A health care professional’s age has an influence on the intention to use eConsulta in the post–COVID-19 context.	Yes	No	No
H7. A health care professional’s gender has an influence on the intention to use eConsulta in the post–COVID-19 context.	Yes	Yes	No
H8. The health care region or zone in which the health care center where a professional works is located has an influence on the intention to use eConsulta in the post–COVID-19 context.	No	No	Yes
H9. The degree of eConsulta implementation at the health care center where a professional works has an influence on the intention to use it in the post–COVID-19 context.	Yes	Yes	No
H10. The ease of use of eConsulta (current version) has an influence on the intention to use it in the post–COVID-19 context.	Yes	Yes	Yes

^a^Not applicable.

## Discussion

### Principal Findings

The objective of this study was to identify the factors that have an influence on eConsulta adoption, and the influence of these and other factors on the intention to use the tool in the post–COVID-19 context. To that end, a theoretical model based on a modified TAM was used as the analysis tool.

eConsulta has become a key tool for providing remote medical care owing to the challenges posed by the COVID-19 pandemic. The use of this tool has increased significantly since the start of the pandemic, and the majority of health care professionals are now able to consider using it in their routine medical practice, even after the relaxation of social distancing measures and a return to some degree of normality. We focused our research on forecasts for tool use in the post–COVID-19 context because, as soon as social distancing measures are removed, it is likely that professionals will have the option to choose how, that is, by which means, they connect with their patients. This possibility to choose, which is not feasible while social distancing measures are in place, is very significant, not only from the perspective of analyzing the explanatory factors of eConsulta use, but also from the perspective of health policies. With the experience of its mass use during lockdown, we analyzed explanatory factors for the future use of the tool within the context of a greater freedom of choice. The purpose of doing so was to find out whether the lockdowns had changed the factors driving the use of digital tools for the provision of medical care or, conversely, whether the health care professionals perceived such use to be exceptional, with their preferences being more aligned with a return to the prepandemic situation. These results are undoubtedly very useful for the design of public policies on health care delivery via digital technologies.

Our study confirms, as previous studies have done [5], that perceived usefulness was the explanatory factor with the biggest effect on the attitude toward and intention to use eConsulta in the post–COVID-19 context. As the TAM suggests, the significance of this determining factor refers back to the importance of perceived usefulness when the use of a technology needs to be explained [46,47]. Specifically, ICS health care professionals placed importance on improved patient relationships and the efficiency of their work in their intention to use the tool, and on perceived benefits in their intention to use the tool in the post–COVID-19 context. In this respect, several studies have shown that telecare reduces the number of low value-added face-to-face visits, thus providing evidence to support intervention efficiency from the health care provider’s viewpoint [48,49]. It is therefore crucial for the health care system’s best way of operating to be accepted by the professionals working within it.

The next most important variable was the type of use that individuals habitually made of eConsulta. Bearing in mind that the core work of physicians is patient care, whereas that of the nurse group involves either management or carrying out tasks related to communicating with, and sending information to, patients, an increase in perceived usefulness by the latter positively influenced their attitude toward, and increased their intention to use, the tool. This is consistent with many studies on the acceptance of telemedicine solutions by both primary care providers [50] and nursing staff [51].

Perceived ease of use also had a positive impact on the attitude toward using eConsulta, in particular on improving some professionals’ attitude toward and intention to use it because they felt that it would not involve any effort; this was especially so in the physician group [30,52]. As seen in previous studies, general practitioners with prior experience in digital health care technologies were more enthusiastic and optimistic than those who had yet to use it [19,49]. Despite the evidence showing that as knowledge of ICT use increases, the difficulties an individual encounters when using it decrease [53], it is worth noting the little weight that this factor had compared to benefits and environmental pressure. This might be due to either the generalized implementation of this tool by the administration or the rapid digital transformation that this sector has experienced as a consequence of COVID-19. However, experience using technological tools was a variable displaying a direct and significant relationship with the intention to use eConsulta in the post–COVID-19 context. This result highlights the importance of developing staff’s competencies for the sustainable adoption of digital solutions in the health care field.

Second in order of importance in the explanation of the tool’s use by ICS health care professionals was the pressure that third parties—work colleagues, patients, or the institution itself—directly or indirectly exerted on them. Environmental pressure also contributed to the intention to continue using eConsulta in the post–COVID-19 context. In this respect, we found that when patients had easy access to the tool, it made them ask health care professionals to use it. Access to the personal health folder, a tool that enables citizens to securely access their personal information and online services [54,55], was key. Similarly, the fact that some colleagues rated the tool positively, or actually used it, also had a direct influence on the intention to use it. This could also be attributed to network effects, which are crucial to the adoption of any technology [56]. Lastly, the fact that the health care institution itself had committed to eConsulta implementation was an important reason for adopting it.

Finally, it should be noted that the degree of eConsulta implementation at the health care center where a professional works had an influence on the intention to use it in the post–COVID-19 context, especially among the physician group. This might be due to the fact that the tool’s implementation differed at each health care center. Regarding nursing staff, a plausible explanation as to why it did not affect them is that each health care region or zone is independent in terms of the types of activity (patient treatment or management) that the nurse group carries out, so the types of use made of the tool differs. In fact, the nurse group was the most reticent in terms of the continued use of the tool in the postlockdown phase. Research into the implications of eHealth and telemedicine on professional practice has repeatedly shown that implementation of digital practices for the provision of medical care leads to significant changes in the tasks that professionals carry out. For a sustainable eConsulta implementation, the tasks that the physician and nurse groups carry out will undoubtedly need to be reviewed to ensure that the provision of value-added health care is more efficient and of higher quality.

The COVID-19 pandemic has led to the implementation of digital solutions at record speed and with unprecedented impact. The experience that this has provided nurses and physicians with increases the likelihood of them continuing to use it in the post–COVID-19 context. It is worth taking advantage of the impetus that the current crisis has given us to implement at least some of the solutions proposed in the scientific literature. This study’s data cannot be extrapolated to other health care systems; however, the results are critical for digital health care policy planners because the success of eConsulta will largely depend on whether health care professionals promote it. In any case, maintaining the drivers of a continued use of digital tools for the provision of medical care must go hand in hand with practices that promote their use by patients. Having professional groups that are active and ready for the digital transformation is of little use if, on the care services demand side, patients continue to opt for face-to-face care as a matter of preference. In this respect, it is important to make further advances in relation to the social dissemination of the strengths of social health care tools, while at the same time putting efforts into reducing their weaknesses, especially the care inequalities that their use may generate.

### Limitations

This study has a number of potential limitations. First, it is a survey-based study, subject to the bias response rates that are inherent to all studies based on data of this type. Second, the survey we used was new and unvalidated, utilized to determine eConsulta use by health care professionals. It is our belief, however, that the survey questions posed were of a pragmatic nature, and that the answers faithfully reflected the sentiments of all the groups. Lastly, the survey was administered in less than 1 month in the midst of drastic changes to medical practice brought about by the pandemic, so opinions and preferences may continue to evolve. Notwithstanding the above, we believe that the sample size analyzed and the degree of statistical significance observed together make our results robust.

### Conclusions

The implementation and use of eConsulta had increased significantly as a consequence of the COVID-19 pandemic, and the majority of the health care professionals were satisfied with its use in practice and planned to incorporate it into their practices in the post–COVID-19 context. Perceived benefits and environmental pressures were determining factors in the attitude toward and intention to use eConsulta. However, some reticence in terms of the continued use of the tool in the post–COVID-19 context was detected, especially among the nurse group. For this digital transformation in health care to continue beyond the pandemic, it is important to establish connections between health care professionals’ use of the tool on one hand and modification of their tasks on the other, and thus improve the quality of their care. In addition, patients must be educated to use the tool more proficiently.

## Appendix

Multimedia Appendix 1 Correlation matrix between variables: overall model.

Multimedia Appendix 2 Tool use by group.

Multimedia Appendix 3 Health care region or zone by group.

## Abbreviations

CFI: comparative fit index
ICS: Institut Català de la Salut
ICT: information and communications technology
NFI: normed fit index
PCT: primary care team
RMSEA: root mean square error of approximation
TAM: Technology Acceptance Model
TLI: Tucker Lewis index
