# Supplementary material for: Determinants of Catalan Public Primary Care Professionals’ Intention to Use Digital Clinical Consultations (eConsulta) in the Post–COVID-19 Context: Mixed Methods Study
Source: J Med Internet Res. 2021 Jun 24;23(6):e28944. doi: 10.2196/28944 (PMC8386368; doi:10.2196/28944)
Supplement: Multimedia Appendix 1 [file jmir_v23i6e28944_app1.docx]

**APPENDIX 1. Correlation matrix between variables: overall model.**

|  | | **1 Age recoded** | **2. gender** | **professional profiles and where they work** | **5. What Catalan Health Institute healthcare region or zone do you work in?** | **7.1 Amount of time he/ she has been working as a healthcare professional** | **V4_F1_perceived usefulness** | **V4_F2 subjective norm** | **V4_F3 experience and ease of use** | **8 eConsulta implementation in the PCT/Service where I work** | **10BI. eConsulta use** |
| --- | --- | --- | --- | --- | --- | --- | --- | --- | --- | --- | --- |
| **1 Age recoded** | **Pearson correlation** | 1 | -0.111^**^ | -0.073^*^ | 0.031 | 0.715^**^ | -0.093^**^ | 0.086^**^ | -0.167^**^ | 0.037 | -0.005 |
|  | **Sig. (bilateral)** |  | 0.000 | 0.011 | 0.281 | 0.000 | 0.001 | 0.003 | 0.000 | 0.204 | 0.875 |
|  | **N** | 1188 | 1188 | 1188 | 1188 | 1188 | 1188 | 1188 | 1188 | 1181 | 1188 |
| **2. gender** | **Pearson correlation** | -0.111^**^ | 1 | 0.170^**^ | 0.023 | -0.044 | 0.064^*^ | 0.073^*^ | -0.018 | 0.041 | -0.074^*^ |
|  | **Sig. (bilateral)** | 0.000 |  | 0.000 | 0.420 | 0.127 | 0.028 | 0.011 | 0.533 | 0.157 | 0.011 |
|  | **N** | 1188 | 1188 | 1188 | 1188 | 1188 | 1188 | 1188 | 1188 | 1181 | 1188 |
| **professional profiles and where they work** | **Pearson correlation** | -0.073^*^ | 0.170^**^ | 1 | -0.005 | -0.051 | -0.040 | -0.082^**^ | 0.075^*^ | -0.058^*^ | -0.463^**^ |
|  | **Sig. (bilateral)** | 0.011 | 0.000 |  | 0.852 | 0.079 | 0.170 | 0.005 | 0.010 | 0.047 | 0.000 |
|  | **N** | 1188 | 1188 | 1188 | 1188 | 1188 | 1188 | 1188 | 1188 | 1181 | 1188 |
| **5. What Catalan Health Institute healthcare region or zone do you work in?** | **Pearson correlation** | 0.031 | 0.023 | -0.005 | 1 | 0.031 | 0.052 | 0.001 | -0.005 | 0.020 | -0.010 |
|  | **Sig. (bilateral)** | 0.281 | 0.420 | 0.852 |  | 0.284 | 0.075 | 0.980 | 0.851 | 0.488 | 0.718 |
|  | **N** | 1188 | 1188 | 1188 | 1188 | 1188 | 1188 | 1188 | 1188 | 1181 | 1188 |
| **7.1_Amount of time he/she has been working as a healthcare professional** | **Pearson correlation** | 0.715^**^ | -0.044 | -0.051 | 0.031 | 1 | -0.027 | 0.072^*^ | -0.147^**^ | 0.023 | 0.014 |
|  | **Sig. (bilateral)** | 0.000 | 0.127 | 0.079 | 0.284 |  | 0.357 | 0.013 | 0.000 | 0.432 | 0.638 |
|  | **N** | 1188 | 1188 | 1188 | 1188 | 1188 | 1188 | 1188 | 1188 | 1181 | 1188 |
| **V4_F1_perceived usefulness** | **Pearson correlation** | -0.093^**^ | 0.064^*^ | -0.040 | 0.052 | -0.027 | 1 | 0.000 | 0.000 | 0.133^**^ | 0.265^**^ |
|  | **Sig. (bilateral)** | 0.001 | 0.028 | 0.170 | 0.075 | 0.357 |  | 0.998 | 0.997 | 0.000 | 0.000 |
|  | **N** | 1188 | 1188 | 1188 | 1188 | 1188 | 1188 | 1188 | 1188 | 1181 | 1188 |
| **V4_F2 subjective norm** | **Pearson correlation** | 0.086^**^ | 0.073^*^ | -0.082^**^ | 0.001 | 0.072^*^ | 0.000 | 1 | 0.000 | 0.156^**^ | 0.207^**^ |
|  | **Sig. (bilateral)** | 0.003 | 0.011 | 0.005 | 0.980 | 0.013 | 0.998 |  | 0.993 | 0.000 | 0.000 |
|  | **N** | 1188 | 1188 | 1188 | 1188 | 1188 | 1188 | 1188 | 1188 | 1181 | 1188 |
| **V4_F3 experience and ease of use** | **Pearson correlation** | -0.167^**^ | -0.018 | 0.075^*^ | -0.005 | -0.147^**^ | 0.000 | 0.000 | 1 | -0.063^*^ | 0.048 |
|  | **Sig. (bilateral)** | 0.000 | 0.533 | 0.010 | 0.851 | 0.000 | 0.997 | 0.993 |  | 0.032 | 0.097 |
|  | **N** | 1188 | 1188 | 1188 | 1188 | 1188 | 1188 | 1188 | 1188 | 1181 | 1188 |
| **8 eConsulta implementation in the PCT/Service where I work** | **Pearson correlation** | 0.037 | 0.041 | -0.058^*^ | 0.020 | 0.023 | 0.133^**^ | 0.156^**^ | -0.063^*^ | 1 | 0.307^**^ |
|  | **Sig. (bilateral)** | 0.204 | 0.157 | 0.047 | 0.488 | 0.432 | 0.000 | 0.000 | 0.032 |  | 0.000 |
|  | **N** | 1181 | 1181 | 1181 | 1181 | 1181 | 1181 | 1181 | 1181 | 1181 | 1181 |
| **10BI. eConsulta use** | **Pearson correlation** | -0.005 | -0.074^*^ | -0.463^**^ | -0.010 | 0.014 | 0.265^**^ | 0.207^**^ | 0.048 | 0.307^**^ | 1 |
|  | **Sig. (bilateral)** | 0.875 | 0.011 | 0.000 | 0.718 | 0.638 | 0.000 | 0.000 | 0.097 | 0.000 |  |
|  | **N** | 1188 | 1188 | 1188 | 1188 | 1188 | 1188 | 1188 | 1188 | 1181 | 1188 |
| **. The correlation is significant at 0.01 (bilateral). | | | | | | | | | | | |
| *. The correlation is significant at 0.05 (bilateral). | | | | | | | | | | | |
