# Supplementary material for: Determinants of Catalan Public Primary Care Professionals’ Intention to Use Digital Clinical Consultations (eConsulta) in the Post–COVID-19 Context: Mixed Methods Study
Source: J Med Internet Res. 2021 Jun 24;23(6):e28944. doi: 10.2196/28944 (PMC8386368; doi:10.2196/28944)
Supplement: Multimedia Appendix 2 [file jmir_v23i6e28944_app2.docx]

**APPENDIX 2. Tool use by group**

| **10BI. eConsulta use** | | | | | | |
| --- | --- | --- | --- | --- | --- | --- |
| **professional profiles and where they work** | | | **Frequency** | **Percent** | **Valid percent** | **Cumulative percent** |
| **.** | **Lost** | **System** | 3 | 100.0 |  |  |
| **general practitioner** | **Valid** | **zero use** | 12 | 2.4 | 2.4 | 2.4 |
|  |  | **management use** | 28 | 5.5 | 5.5 | 7.9 |
|  |  | **wide use consultation** | 467 | 92.1 | 92.1 | 100.0 |
|  |  | **Total** | 507 | 100.0 | 100.0 |  |
| **paediatrician** | **Valid** | **zero use** | 17 | 9.3 | 9.3 | 9.3 |
|  |  | **management use** | 39 | 21.4 | 21.4 | 30.8 |
|  |  | **wide use consultation** | 126 | 69.2 | 69.2 | 100.0 |
|  |  | **Total** | 182 | 100.0 | 100.0 |  |
| **family nurse** | **Valid** | **zero use** | 58 | 19.5 | 19.5 | 19.5 |
|  |  | **management use** | 89 | 30.0 | 30.0 | 49.5 |
|  |  | **wide use consultation** | 150 | 50.5 | 50.5 | 100.0 |
|  |  | **Total** | 297 | 100.0 | 100.0 |  |
| **sexual health service nurse** | **Valid** | **zero use** | 73 | 36.0 | 36.0 | 36.0 |
|  |  | **management use** | 45 | 22.2 | 22.2 | 58.1 |
|  |  | **wide use consultation** | 85 | 41.9 | 41.9 | 100.0 |
|  |  | **Total** | 203 | 100.0 | 100.0 |  |
