# Supplementary material for: Determinants of Catalan Public Primary Care Professionals’ Intention to Use Digital Clinical Consultations (eConsulta) in the Post–COVID-19 Context: Mixed Methods Study
Source: J Med Internet Res. 2021 Jun 24;23(6):e28944. doi: 10.2196/28944 (PMC8386368; doi:10.2196/28944)
Supplement: Multimedia Appendix 3 [file jmir_v23i6e28944_app3.docx]

**APPENDIX 3. Healthcare region or zone by group.**

| **What Catalan Health Institute healthcare region or zone do you work in?** | | | | | | |
| --- | --- | --- | --- | --- | --- | --- |
| **professional profiles and where they work** | | | **Frequency** | **Percent** | **Valid percent** | **Cumulative percent** |
| **.** | **Lost** | **System** | 3 | 100.0 |  |  |
| **general practitioner** | **Valid** | **Metropolitan North** | 100 | 19.7 | 19.7 | 19.7 |
|  |  | **Central Catalonia** | 76 | 15.0 | 15.0 | 34.7 |
|  |  | **Barcelona City** | 110 | 21.7 | 21.7 | 56.4 |
|  |  | **Lleida** | 21 | 4.1 | 4.1 | 60.6 |
|  |  | **Metropolitan South** | 67 | 13.2 | 13.2 | 73.8 |
|  |  | **High Pyrenees and Aran** | 1 | 0.2 | 0.2 | 74.0 |
|  |  | **Girona** | 83 | 16.4 | 16.4 | 90.3 |
|  |  | **Camp de Tarragona** | 35 | 6.9 | 6.9 | 97.2 |
|  |  | **Terres de l’Ebre** | 14 | 2.8 | 2.8 | 100.0 |
|  |  | **Total** | 507 | 100.0 | 100.0 |  |
| **paediatrician** | **Valid** | **Metropolitan North** | 40 | 22.0 | 22.0 | 22.0 |
|  |  | **Central Catalonia** | 26 | 14.3 | 14.3 | 36.3 |
|  |  | **Barcelona City** | 28 | 15.4 | 15.4 | 51.6 |
|  |  | **Lleida** | 9 | 4.9 | 4.9 | 56.6 |
|  |  | **Metropolitan South** | 8 | 4.4 | 4.4 | 61.0 |
|  |  | **High Pyrenees and Aran** | 2 | 1.1 | 1.1 | 62.1 |
|  |  | **Girona** | 52 | 28.6 | 28.6 | 90.7 |
|  |  | **Camp de Tarragona** | 16 | 8.8 | 8.8 | 99.5 |
|  |  | **Terres de l’Ebre** | 1 | 0.5 | 0.5 | 100.0 |
|  |  | **Total** | 182 | 100.0 | 100.0 |  |
| **family nurse** | **Valid** | **Metropolitan North** | 45 | 15.2 | 15.2 | 15.2 |
|  |  | **Central Catalonia** | 39 | 13.1 | 13.1 | 28.3 |
|  |  | **Barcelona City** | 56 | 18.9 | 18.9 | 47.1 |
|  |  | **Lleida** | 12 | 4.0 | 4.0 | 51.2 |
|  |  | **Metropolitan South** | 38 | 12.8 | 12.8 | 64.0 |
|  |  | **Girona** | 58 | 19.5 | 19.5 | 83.5 |
|  |  | **Camp de Tarragona** | 24 | 8.1 | 8.1 | 91.6 |
|  |  | **Terres de l’Ebre** | 25 | 8.4 | 8.4 | 100.0 |
|  |  | **Total** | 297 | 100.0 | 100.0 |  |
| **sexual health service nurse** | **Valid** | **Metropolitan North** | 65 | 32.0 | 32.0 | 32.0 |
|  |  | **Central Catalonia** | 33 | 16.3 | 16.3 | 48.3 |
|  |  | **Barcelona City** | 24 | 11.8 | 11.8 | 60.1 |
|  |  | **Lleida** | 9 | 4.4 | 4.4 | 64.5 |
|  |  | **Metropolitan South** | 20 | 9.9 | 9.9 | 74.4 |
|  |  | **High Pyrenees and Aran** | 2 | 1.0 | 1.0 | 75.4 |
|  |  | **Girona** | 20 | 9.9 | 9.9 | 85.2 |
|  |  | **Camp de Tarragona** | 19 | 9.4 | 9.4 | 94.6 |
|  |  | **Terres de l’Ebre** | 11 | 5.4 | 5.4 | 100.0 |
|  |  | **Total** | 203 | 100.0 | 100.0 |  |
